# Changes of IgG *N*-Glycosylation in Thyroid Autoimmunity: The Modulatory Effect of Methimazole in Graves’ Disease and the Association With the Severity of Inflammation in Hashimoto’s Thyroiditis

**DOI:** 10.3389/fimmu.2022.841710

**Published:** 2022-03-15

**Authors:** Sara Trzos, Paweł Link-Lenczowski, Grzegorz Sokołowski, Ewa Pocheć

**Affiliations:** ^1^ Department of Glycoconjugate Biochemistry, Institute of Zoology and Biomedical Research, Faculty of Biology, Jagiellonian University, Kraków, Poland; ^2^ Department of Medical Physiology, Faculty of Health Sciences, Jagiellonian University Medical College, Kraków, Poland; ^3^ Department of Endocrinology, Faculty of Medicine, Jagiellonian University Medical College, Kraków, Poland

**Keywords:** immunoglobulin G (IgG), N-glycosylation, Graves’ disease (GD), Hashimoto’s thyroiditis (HT), immunosuppressive therapy, ultraperformance liquid chromatography - mass spectrometry (UPLC-MS)

## Abstract

The *N*-glycome of immunoglobulin G (IgG), the most abundant glycoprotein in human blood serum, reflects pathological conditions of autoimmunity and is sensitive to medicines applied in disease therapy. Due to the high sensitivity of *N*-glycosylation, the IgG *N*-glycan profile may serve as an indicator of an ongoing inflammatory process. The IgG structure and its effector functions are strongly dependent on the composition of *N*-glycans attached to the Fc fragment, and the binding of antigens is regulated by Fab sugar moieties. Because of the crucial role of *N*-glycans in IgG function, remodeling of its *N*-oligosaccharides can induce pathological changes that ultimately contribute to the development of autoimmunity; restoration of their physiological structure is critical to the reduction of disease symptoms. Our recently published data have shown that the pathology of autoimmune thyroid diseases (AITDs), including Hashimoto’s thyroiditis (HT) and Graves’ disease (GD), is accompanied by alterations of the composition of IgG *N*-glycans. The present study is a more in-depth investigation of IgG glycosylation in both AITDs, designed to determine the relationship between the severity of thyroid inflammation and IgG *N*-glycan structures in HT, and to assess the impact of immunosuppressive therapy on the *N*-glycan profile in GD patients. The study material consisted of human serum samples collected from donors with elevated anti-thyroglobulin (Tg) and/or anti-thyroperoxidase (TPO) IgGs without symptoms of hypothyroidism (n=68), HT patients characterized by high autoantibody titers and advanced destruction of the thyroid gland (n=113), GD patients with up-regulated IgG against thyroid-stimulating hormone receptor (TSHR) before (n=62) and after (n=47) stabilization of TSH level as a result of methimazole therapy (study groups), and healthy donors (control group, n=90). IgG was isolated from blood serum using protein G affinity chromatography. *N*-glycans were released from IgG by PNGase F digestion and analyzed by ultra-performance liquid chromatography-mass spectrometry (UPLC-MS) after 2-aminobenzamide (2-AB) labeling. UPLC-MS chromatograms were integrated into 25 peaks (GP) in the Waters UNIFI Scientific Information System, and *N*-glycans were assigned based on the glucose unit values and mass-to-charge ratios (m/z) of the detected ions. The Kruskal-Wallis non-parametric test was used to determine the statistical significance of the results (p<0.05). The obtained results suggest that modifications of IgG sialylation, galactosylation and core-fucosylation are associated with the severity of HT symptoms. Methimazole therapy implemented in GD patients affected the IgG *N*-glycan profile; as a result, the content of the sialylated and galactosylated oligosaccharides with core fucose differed after treatment. Our results suggest that *N*-glycosylation of IgG undergoes dynamic changes during the intensification of thyroiditis in HT, and that in GD autoimmunity it is affected significantly by immunosuppressive therapy.

## 1 Introduction

Class G immunoglobulins (IgGs), produced by plasma cells and B cells, are the most abundant human serum glycoproteins. They are an essential component of humoral immune responses and are involved in the recognition, neutralization, and elimination of pathogens and toxic antigens (1). The antigen-binding fragment (Fab) of IgG binds specifically antigens, while the crystallizable fragment (Fc) is responsible for mediating the antibody effector functions. Due to its great importance in immune response, IgG is one of the best-studied glycoproteins, i.e. proteins modified post-translationally by covalent attachment of various oligosaccharide structures, called glycans (2, 3).

Oligosaccharides represent up to 15% of IgG molecular weight (4). They are attached to both Fc and Fab fragments and belong to the *N*-glycans characterized by the presence of an *N*-glycosidic bond linking an *N*-acetylglucosamine (GlcNAc) in sugar structure with asparagine (Asn) within the Asn-X-Ser/Thr amino acid sequence. Each IgG molecule has two highly conserved *N*-glycosylation sites located at Asn297 within the CH2 domains of the Fc fragment (5–7). Mature IgG Fc glycoforms possess mainly diantennary complex-type structures differing in the number of monosaccharides building the antennae, namely GlcNAc, galactose (Gal), and sialic acid (SA) as well as in the number of bisecting GlcNAc residue, and the presence of core fucose (Fuc) (8, 9). Fab fragments have also been found to be *N*-glycosylated but only in 10-30% of IgGs (7, 10, 11). It has been demonstrated that altering the oligosaccharide composition in the IgG Fc region affects its secretion, tertiary structure, half-life, and effector functions (10, 12–15). Interestingly, a complete deletion of Fc *N*-glycans results in loss of pro- and anti-inflammatory IgG activity (16).

The composition of the IgG glycome is influenced by both genetic and environmental factors, making it an excellent biomarker of overall human health. It seems likely that the IgG *N*-glycosylation process in healthy individuals undergoes little change during homeostasis, while its disruption may be influenced by sex hormones, age, and stress (6, 17, 18). Furthermore, changes in IgG *N*-oligosaccharide patterns have been implicated in disease progression and remission, representing both predisposition and functional mechanisms involved in the pathogenesis of diseases, including autoimmune diseases (6, 19). Some of the most common autoimmune disorders are autoimmune thyroid diseases (AITDs), which include Graves’ disease (GD) and Hashimoto’s thyroiditis (HT) (20).

The development of AITD follows a loss of immune tolerance and reactivity to thyroid autoantigens, leading to infiltration of thyroid gland by T cells and B cells that produce characteristic antibodies. An activity of autoantibodies against thyrotropic hormone receptor (TSHR) expressed on thyroid follicular cells (thyrocytes) initiates the development of GD. Intense stimulation of TSHR by anti-TSHR antibodies (TRAb) leads to increased secretion of thyroid hormones, thyroxine (T4) and triiodothyronine (T3), resulting in hyperthyroidism. As a result, GD-associated autoimmunity can cause goiter, ophthalmopathy or thyroid dermopathy. TSHR activation by anti-TSHR can also stimulate the growth of thyrocytes and causes the development of thyroid vascularization (21). In the HT immune tolerance to thyroid self-antigens is lost, resulting in the destruction of thyrocytes by activated T lymphocytes which leads to hypothyroidism. HT is diagnosed based on the elevated levels of circulating antibodies against thyroid antigens: thyroperoxidase (TPO) and thyroglobulin (Tg), and is supported by the decreased thyroid echogenicity on ultrasound (22). In HT patients, a Th1 immune response predominates, promoting cellular immunity and thyroid follicular apoptosis (23, 24). Proapoptotic ligands and death receptors such as FasL, TNF and TRAIL, present on thyrocytes, remain inactive under physiological conditions (23). Fas/FasL expression induced in Th1 response by infiltration of pro-inflammatory cytokines TNF-α and IL-1β activates thyroid follicular cell apoptosis in HT (24). In GD, the predominance of Th2 cells promotes a humoral response, with increased production of TRAb antibodies by B cells. The presence of autoantibodies and the increased level of cytokines produced by Th2 lymphocytes, inhibits Fas/FasL expression and results in the activation of anti-apoptotic molecule Bcl-2, which protects thyroid cells from apoptosis, but increases the death of T cells infiltrating the gland tissue (25, 26). Apart from apoptosis, thyroid cells are destroyed by anti-TPO IgG, present in serum of about 80-95% of patients (27). Anti-TPO IgG is involved in thyrocyte destruction through antibody-dependent cellular cytotoxicity (ADCC) (28, 29) and complement-dependent cytotoxicity (CDC) (29). Environmental factors, mainly nuclear radiation, iodine, smoking, infections, stress, alcohol and drugs, contribute approximately to 20% of all AITDs (30). Genetic factors are also implicated in the development of diseases. Studies conducted over many years have identified several genes and chromosome regions that are associated with the development of GD and HT (30, 31).

Changes in IgG *N*-glycans have been demonstrated in chronic inflammation, including autoimmune diseases. The altered *N*-glycan profile of IgG has been proven to be a valuable indicator of systemic lupus erythematosus (SLE) and rheumatoid arthritis (RA) (32, 33). Reduced galactosylation of IgG in RA, described for the first time by Parekh et al. (1985), has been repeatedly confirmed in subsequent analyses by numerous research groups (32, 34, 35). Currently it is the best-characterized modification of IgG glycosylation, used as a serum glycomarker of RA progression and remission (36). Alteration of α1,6-fucosylation is, after agalactosylation, one of the most common modifications of IgG *N*-glycans observed in inflammatory diseases (37). The vast majority of human IgG Fc glycoforms (over 90%) are core fucosylated (6, 34), and this modification plays a crucial role in the modulation of IgG biological activity. Up-regulation of *N*-glycans with core Fuc on IgG heavy chains has been demonstrated in RA patients compared to healthy individuals (38) and the higher exposure of Fuc residues on IgG *N*-oligosaccharides has been shown in the blood of SLE donors than in healthy people (19). Reduced α1,6-fucosylation has been observed in SLE IgG during remission compared to patients with acute SLE (19). The further study performed with the use of the UPLC method showed a decreased galactosylation, sialylation, and core fucosylation of IgG accompanied by an increase of bisecting GlcNAc in IgG *N*-glycans from three independent populations of SLE patients in comparison to healthy donors (39). Our recent study performed on three European cohorts revealed a reduced core fucosylation of IgG in AITD patients compared to a population of healthy donors and a correlation of this IgG modification with anti-TPO serum titer (40). Terminally located sialic acid is another monosaccharide building IgG *N*-glycans that fundamentally regulates the activity of this glycoprotein. Down-regulation of IgG sialylation, which is usually followed by agalactosylation in inflammatory conditions, significantly promotes the pro-inflammatory potential of antibodies (6, 36).

The mechanisms underlying the impact of IgG Fc *N*-glycan fucosylation and sialylation on this protein physiological property have been widely described. Several studies have demonstrated for example the higher cytotoxicity in antibody-dependent cell-mediated (ADCC) and complement-dependent cytotoxicity (CDC) mediated by IgG with reduced sialylation and fucosylation (41–43). Attachment of SA to glycans affects the structure of the Fc fragment in IgG and leads to a 10-fold reduction in the affinity of antibodies for FcγR, thereby impairing their effector functions (44). Increased sialylation reduces cytotoxic activity in ADCC (45). The inhibitory effect of sialic acid in IgG *N*-oligosaccharides on ADCC may be due to reduced binding of IgG to its receptor as a result of impaired antigen binding caused by the lack of flexibility of the IgG hinge region (46). Fucosylation of the core of IgG *N*-glycans is also crucial for the control of ADCC, as IgG lacking α1,6-linked Fuc on Fc has been found to have up to 100-fold increased ADCC activity (42).

Until recently, it was thought that each step of *N*-glycosylation could only occur intracellularly in the rough endoplasmic reticulum (RER) and Golgi apparatus (GA). However, it has been found that α2,6-sialylation can also occur in the bloodstream in a reaction catalyzed by β-galactoside α2,6-sialyltransferase 1 (ST6Gal1) which transfers sialic acid from nucleotide sugar donor cytidine monophosphate (CMP)-SA, secreted by platelet α granules, to Gal in IgG *N*-glycan and creates between them α2,6-glycosidic bond. ST6Gal1 is produced by hepatic central veins and secreted into the bloodstream. This discovery sheds new light on the process of IgG sialylation and may explain the great dynamics of inflammatory processes mediated by antibodies (47).

*N*-glycans on IgG are rebuilt not only during disease development and progression, but their composition is also sensitive to medications used during the treatment of patients, including immunosuppressive drugs. Up-regulation of IgG galactosylation was observed in patients with RA who have been treated with methotrexate (48–50) and infliximab (chimeric anti-tumour necrosis factor α monoclonal antibodies, anti-TNFα IgG) (51). Moreover, restoration of *N*-glycan galactosylation correlated with significant clinical improvement following infliximab therapy (51). Nonsteroidal anti-inflammatory drugs (NSAIDs), i.e. aspirin, and also glucocorticosteroids, were shown to influence the glycosylation of human plasma proteins. NSAIDs and corticosteroids reduced core-fucosylation of di- and triantennary complex-type structures, together with oligomannose oligosaccharide levels in plasma *N*-glycome (52).

Our recently published study showed the changes of IgG *N*-glycome are characteristic for AITD patients (40). The present study aimed to investigate the IgG *N*-glycosylation in thyroid autoimmunity in more depth by determining the relationship between thyroiditis severity and IgG *N*-glycan structure in HT and by evaluating the effect of methimazole therapy on the *N*-oligosaccharide profile in GD patients. A comparative analysis of the IgG *N*-glycome was performed by ultra-performance liquid chromatography combined with mass spectrometry (UPLC-MS). We also examined B-cell independent α2,6-sialylation of IgG in AITD blood sera considering it as a particularly interesting mechanism of *N*-glycan modification.

## 2 Materials and Methods

### 2.1 Bioethical Statement

The study was conducted in accordance with the Declaration of Helsinki and approved by the Bioethics Committee of the Jagiellonian University in Krakow, Poland (1072.6120.99.2021). All individuals donating blood for the experiment signed informed consent. Blood samples were collected between May 2014 and March 2021.

### 2.2 Characteristics of the Study Groups

The study was conducted in cooperation with the Department of Endocrinology of the Jagiellonian University Hospital in Krakow. Recruitment of adult patients with AITDs without any other concomitant autoimmune diseases, and healthy volunteers was performed by an endocrinologist based on thyrotropic hormone (TSH) and thyroid autoantibody levels, thyroid gland ultrasonography and medical history of donors. Patients with AITD affected by other diseases, taking other drugs than those associated with AITD, donors suffering from alcoholism and taking other stimulants as well as pregnant women were excluded.

**Figure 1:**
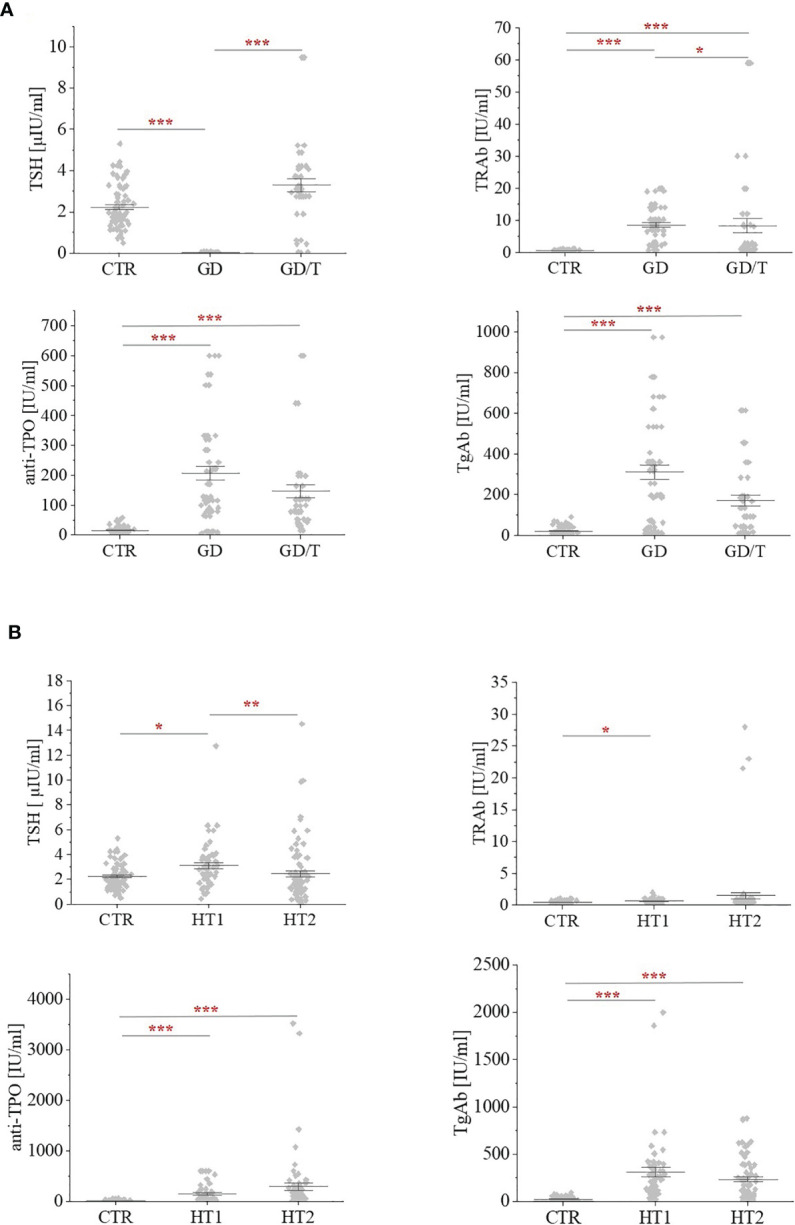
The levels of thyrotropic hormone (TSH) and autoantibodies: against the receptor for thyrotropin (TRAb); anti-thyroperoxidase (anti-TPO); thyroglobulin antibodies (TgAb) within **(A)** Graves’ disease groups: before (GD) and after (GD/T) TSH normalization, and **(B)** Hashimoto’s thyroiditis groups: the donors with the elevated TgAb and/or anti-TPO (HT1), Hashimoto’s thyroiditis patients (HT2) relative to the control group (CTR). Statistically significant differences were determined at p < 0.05 (*), p < 0.01 (**), and p < 0.001 (***).

Human serum samples were collected from donors with the elevated IgG anti-thyroglobulin (TgAb) and/or anti-thyroperoxidase (anti-TPO) levels without symptoms of hypothyroidism (HT1, n=68), from Hashimoto’s thyroiditis patients characterized by the high autoantibody titers, advanced thyroid destruction and with the stabilized TSH level as a result of L-thyroxine treatment (HT2, n=113), GD patients with the elevated thyrotropic hormone receptor IgG (TRAb) levels before (GD, n=62) and after (GD/T, n=47) stabilization of TSH level as a result of the treatment with methimazole (1-methyl-2-mercaptoimidazole), an anti-thyroid agent with immunosuppressive activity. Healthy donors with the serum titers of TSH and autoantibodies within the normal range constituted a control group (CTR, n=90). The control group was age-matched to the study groups. **Table 1** contains the demographic and clinical characteristics of donors with AITDs and healthy volunteers, and **Figure 1** shows a graphical comparison of TSH and autoantibody (anti-TPO, TgAb, and TRAb) levels between the groups.

**Table 1:** Characteristics of healthy donors (CTR, control group) and patients with elevated TgAb and/or anti-TPO levels without symptoms of hypothyroidism (HT1), patients with Hashimoto’s thyroiditis (HT2) and patients with Graves’ disease before (GD) and after (GD/T) TSH normalization (study groups).

status	age	sex	TSH	TRAb	anti-TPO	TgAb
		(F/M)	0.27-4.20 µIU/ml	0.0-1.0 IU/ml	<34.0 IU/ml	<115.0 IU/ml
**CTR**	18-64	67/8	2.22±1.04	0.47±0.26	19.60±18.54	10.63±1.55
	34±8.75					
**HT1**	18-66	53/2	3.10±1.93	0.63±0.36	140.78±174.24	310.22±358.76
	35±10.55					
**HT2**	20-62	88/0	2.46±2.34	1.49±4.61	294.57±605.09	233.32±231.21
	36±10.17					
**GD**	22-55	46/15	0.01±0.02	8.47±5.90	207.33±175.61	311.37±270.29
	35±9.08					
**GD/T**	23-56	33/13	3.29±2.18	8.27±15.31	146.26±149.66	170.12±184.02
	37±9.46					

Mean ± SD is given for age, TSH and antibody levels. anti-TPO, anti-thyroperoxidase antibodies; TgAb, thyroglobulin antibodies; TSH, thyrotropic hormone; TRAb, antibodies directed against the receptor for thyrotropin; F, female; M, male.

### 2.3 Collection of Serum Samples

Blood samples were collected by venipuncture into the tubes containing a clotting activator (S-Monovette, Sarstedt, 04.1934.001), left for 5 h at room temperature (RT) for blood clotting, and centrifuged at 2500 rpm for 10 min at 4°C (Heraeus). Serum samples were stored at -80°C until IgG isolation.

### 2.4 IgG Purification

IgGs were isolated by affinity chromatography with G-protein conjugated to the deposit grains. Serum samples (100 µl) were diluted 1:1 with a binding buffer (0.1 M Na_2_HPO_4_, 0.15 M NaCl, pH 7.2), applied to 96-well Protein G Spin Plates (Thermo Fisher Scientific, 45204), and incubated for 30 min at RT on an orbital shaker (Biosan). After incubation, the plates were centrifuged (1000 rpm, 1 min, RT) and the fraction containing IgG-depleted serum was discarded. Agarose resin was washed with the binding buffer until the ballast proteins were removed which was monitored by measuring the protein absorbance at 280 nm against the binding buffer (NanoDrop 2000, Thermo Fisher Scientific). Then IgGs were eluted with 200 µl of 0.1 M glycine, pH 2.5 into the collection plates containing 20 µl per well of a neutralization buffer (1 M Tris). IgG concentration was determined at 280 nm against a reagent blank (NanoDrop 2000, Thermo Fisher Scientific). IgG samples were stored at -80°C for further analyses.

The efficiency of IgG isolation from human serum was evaluated by SDS-PAGE. Purified IgG (10 µl) was separated on 10% gels under reducing conditions and then stained with colloidal Coomassie Brilliant Blue G-250 (CBB) (Sigma-Aldrich, B2025) according to the manufacturer’s protocol. The molecular masses of the light (25 kDa) and heavy chains (50 kDa) were verified using PageRuler™ Prestained Protein Ladder (Thermo Fisher Scientific, 26616).

### 2.5 Sample Preparation for UPLC-MS Analysis

#### 2.5.1 Buffer Exchange After IgG Isolation

Before deglycosylation, the glycine buffer was exchanged for ammonium formate (AmF) buffer using an Acroprep Advance 96 10 MWCO filter plate (Pall Corporation). The membrane of the plate was prewashed three times with 100 µl of deionized water (Milli-Q, Millipore) by filtration on a microplate vacuum manifold (Waters). After the last wash, 50 µg or 100 µg of IgG was applied to the wells followed by the addition of 100 µl of 100 mM AmF buffer pH 7.5, and the solution was filtered to a minimum volume. The plate was then washed three times with 100 µl of AmF buffer by filtration. Finally, 25 µl AmF buffer was added to the wells and the samples were transferred by pipetting to a PCR plate (Thermo Fisher Scientific), which was repeated twice for each well to maximize the recovery of the IgGs. Finally, the samples were lyophilized to dryness (Labconco) and stored at -80°C.

#### 2.5.2 Enzymatic De-*N*-Glycosylation of IgG

Samples after lyophilization were resuspended in 19 µl of Rapid PNGase F buffer (New England Biolabs). They were then denatured for 15 min at 80°C. After cooling the plate to RT, 0.9 µl of Rapid PNGase F (New England Biolabs, P0710S) was added to each well and the plate was incubated for 25 min at 50°C. After deglycosylation, samples were diluted with 100 µl Milli-Q. IgG-released *N*-glycans were desalted by solid-phase extraction on HyperSepTM Hypercarb™ SPE 96-Well Plates with 10 mg bed weight (Thermo Fisher Scientific, 60302-606). The plate was prewashed three times with 400 µl of 80% acetonitrile (AcN) with 0.1% trifluoroacetic acid (TFA) and then three times with 400 µl of Milli-Q, on the vacuum filtration manifold. The samples were then applied and the columns were washed three times with 400 µl of Milli-Q. In the next step, *N*-oligosaccharides were eluted with 25% AcN + 0.05% TFA into a 96-well Sample Collection Plate (Waters, 186005837). The collected fractions were dried-down by lyophilization (Labconco).

#### 2.5.3 Fluorescent Labeling of *N*-Glycans

*N*-glycan labeling with 2-aminobenzamide (2-AB) was performed according to the procedure previously described by Link-Lenczowski et al. (53). Briefly, the labeling solution consisting of 2-AB (60 mg/mL, Sigma-Aldrich, A89804) and sodium cyanoborohydride (60 mg/mL, Fluka, 156159) in acetic acid:DMSO (3:5) was added to the lyophilized *N*-glycan samples and incubated for 3 h at 65°C. After cooling the plate to RT, the excess dye was removed on a BioZen *N*-Glycan Clean-Up Microelution Plate (Phenomenex, 8M-S009-NGA) according to the manufacturer’s instructions. Elution of labeled sugar structures was performed using 200 mM ammonium acetate in AcN:water (5:95). Eluted glycan samples were lyophilized (Labconco) and stored at -80°C for further analysis.

#### 2.5.4 UPLC-MS Analysis

2-AB labeled *N*-glycans were analyzed by liquid chromatography-mass spectrometry (LC-MS) on Aquity I-Class Plus UPLC system with an in-line fluorescent detector coupled through electrospray ion source to the Vion^®^ IMS-QToF high-resolution mass spectrometer (Waters). *N*-glycans were loaded in 50% AcN and separated by HILIC-UPLC on ACQUITY UPLC Glycan BEH Amide Column, 130Å, 1.7 µm, 2.1 mm X 150 mm (Waters) at 60°C with the following gradient conditions: solvent A was 50 mM ammonium formate pH 4.4, solvent B was 100% acetonitrile (Chemsolv), time = 0 min (t = 0.0), 25% A, 0.4 mL/min; t = 35.0, 46% A, 0.4 mL/min; t = 36.0, 100% A, 0.2 mL/min; t = 39.5, 100% A, 0.2 mL/min; t = 43.5, 25% A, 0.2 mL/min; t = 47.6, 25% A, 0.4 mL/min; t = 55.0, 25% A, 0.4 mL/min. The fluorescence detector was set at Exλ 330 nm and Emλ 420 nm and for electrospray ionization the following parameters were used: capillary voltage: 3.0 kV, source temperature: 120°C, desolvation temperature 350°C, desolvation gas flow: 800 L/h. The mass spectrometer was operated in positive ion ToF MS mode and the ions from m/z 600 to m/z 2000 were registered with the mass correction by sampling the reference mass standard once every 60 seconds. The UPLC runs were externally calibrated with a 2-AB-labeled glucose homopolymer standard (Waters, 186006841). The resulting chromatograms were automatically integrated with manual correction into 25 peaks to which glycans were assigned based on glucose unit (GU) values and exact mass. The chromatographic and mass data were analyzed with the use of the Waters UNIFI scientific information system with the integrated Waters Glycan GU Scientific Library.

### 2.6 Statistical Analysis

Based on the relative intensity of the glycan peaks (GPs), expressed as percent (%) of the total area of all GPs, statistical analysis was performed using the Kruskal-Wallis nonparametric test with significance at p<0.05. All statistical interpretations of the results were performed in Origin Pro 2021b software (Origin Lab).

### 2.7 Evaluation of Serum ST6Gal1 Activity Using Desialylated IgG

To assess the catalytic activity of serum ST6Gal1, desialylated IgG heavy chains immobilized on PVDF membrane were incubated with human sera from control and study groups according to the protocol by Jones et al. (47) with the minor modifications. The purified IgGs were digested with *Arthrobacter ureafaciens* sialidase with a wide specificity (ABS, 1 U in 100 mL, Roche, 10269611001) to remove sialic acid (SA) from the antibody *N*-glycans. IgG samples (0.5 μg) were incubated with 1 μl of ABS in 8 μl of 50 mM sodium acetate, pH 5.2 overnight at 37°C (Biosan thermoblock). Desialylated and untreated IgG samples were separated under reducing conditions on 10% gels in SDS-PAGE, electrotransferred onto a PVDF membrane (Millipore, 88518), which was cut into small pieces containing IgG heavy chains based on PageRuler™ Prestained Protein Ladder (Thermo Fisher Scientific, 26616), and blocked overnight in 1% BSA (Sigma-Aldrich, A7906) at 4°C. The selected PVDF sections were incubated with human sera from each study and control group diluted 1:1 with TBST (50 mM Tris-HCl, 150 mM NaCl, and 10% Tween) overnight at 4°C. Then the membranes were probed 1 h at RT with biotinylated *Sambucus nigra* agglutinin (SNA, Vector Lab., B-1305) diluted 1:4000 in TBS containing 0.1 mM CaCl_2_ and 0.01 mM MgCl_2_ or with SNA preincubated with 1 M acetic acid as a negative control to check nonspecific binding of SNA *via* protein domains but not to α2,6-sialylated *N*-glycans. After washing three times with TBST, alkaline phosphatase (AP)-conjugated avidin (Sigma-Aldrich, E2636) diluted 1:4000 in TBST was applied for 1 h at RT. IgG heavy chain was visualized by a colorimetric reaction using 5-bromo-4-chloro-3-indolylphosphate (BCIP, Roche, 11383221001) and nitro-blue-tetrazolium (NBT, Roche, 11383213001).

ABS re-digestion of IgG *N*-glycans sialylated by serum ST6Gal1 on PVDF membrane was used as an additional control. Membrane fragments with IgG heavy chains sialylated by serum ST6Gal1 were placed in 4-well plates (Nunc, 176740), and incubated with 3 μl of ABS in 27 μl of 50 mM sodium acetate, pH 5.2 overnight at 37°C (Forma Steri-Cycle i160 CO_2_ incubator, Thermo Fisher Scientific). Then the membranes have been subjected to incubation with SNA and visualized by colorimetric reaction as described above.

## 3 Results

The analysis of *N*-glycosylation was performed on IgG purified from human serum samples according to standard protocol with agarose-linked protein G as a ligand for IgG capturing. IgG was isolated from the sera of patients with Graves’ disease before and after normalisation of TSH level as the result of thyrostatic therapy, the donors with the elevated antithyroid Abs without hypothyroidism, and the patients with Hashimoto’s thyroiditis (GD, GD/T, HT1, and HT2 respectively). The control group consisted of healthy individuals (CTR). The characteristic of healthy donors and patients with AITDs is shown in **Table 1** and **Figure 1**. As expected, TSH level was significantly down-regulated in GD patients in relation to CTR and normalised during methimazole therapy. TRAb serum level, an immunological marker of Graves’ disease, was markedly increased in GD and partially normalized during therapy. GD donors showed also enhanced anti-TPO and TgAb titers, which remained elevated during treatment (**Table 1** and **Figure 1A**). The level of TSH was significantly higher in HT1 than CTR, but still within the normal range and these donors recruited to HT1 group did not show hypothyroidism, like the patients with Hashimoto’s thyroiditis. Anti-TPO and TgAb levels were significantly up-regulated in both HT1 and HT2 donors (**Table 1** and **Figure 1B**).

To verify the efficiency of IgG isolation, the eluted samples were resolved on SDS-PAGE under reducing conditions and IgG chains were visualised by CBB staining. CBB profiles showed the highest intensity of the bands corresponding to IgG heavy chain of ca. 50 kDa and a light chain of about 25 kDa. The staining revealed also the presence of a small portion of other proteins in the eluates, but with much weaker staining intensities than IgG chains, indicating their non-specific binding to the G protein which was also previously shown by Croce et al., who estimated IgG purity to be higher than 90% (54). CBB staining of the eluted proteins is shown in **Supplementary Figure 1**.

**Figure 2:**
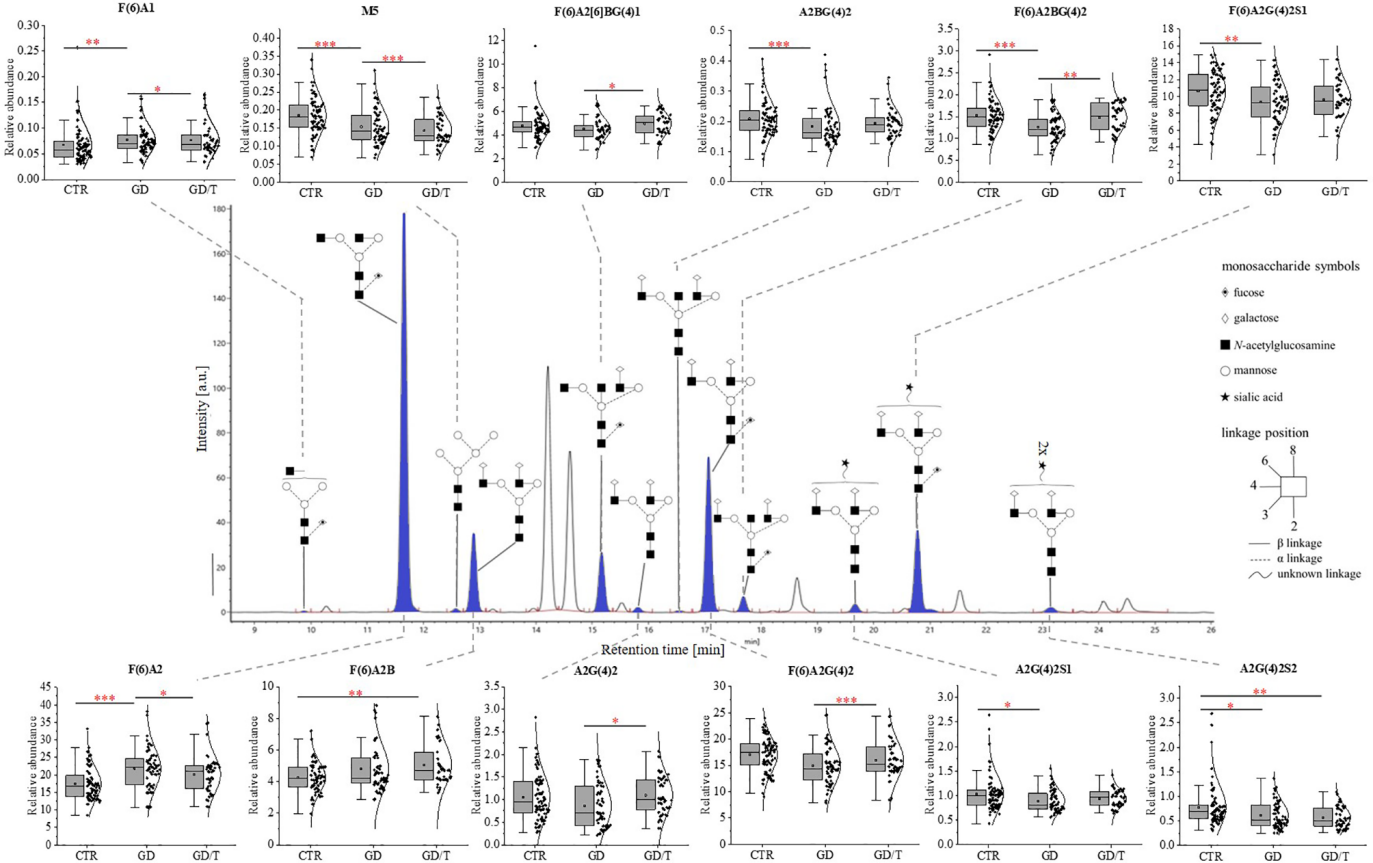
The changes in the content of IgG *N*-glycans from patients with Graves’ disease before (GD, n = 62) and after (GD/T, n = 47) stabilization of TSH level as a result of the treatment with methimazole, and the control group (CTR, n = 90). The chromatograms obtained by UPLC-MS from the separation of IgG *N*-glycans were manually integrated into 25 glycan peaks (GPs) and *N*-oligosaccharide structures were assigned based on GU values and m/z ratios using Waters UNIFI scientific information system software. *N*-glycan structures in Oxford notation were drawn in Sugar Bind software (https://sugarbind.expasy.org/). GPs with statistically significant differences in the content of sugar structures between the study groups (GD, GD/T) and the control group (CTR) are indicated in blue. Statistical analysis was performed using the Kruskal-Wallis non-parametric test, p < 0.05 (*), p < 0.01 (**), and p < 0.001 (***). A1-2, number of antennas; B, bisecting *N*-acetylglucosamine; F, fucose; G, galactose, M, mannose; S, sialic acid.

**Figure 3:**
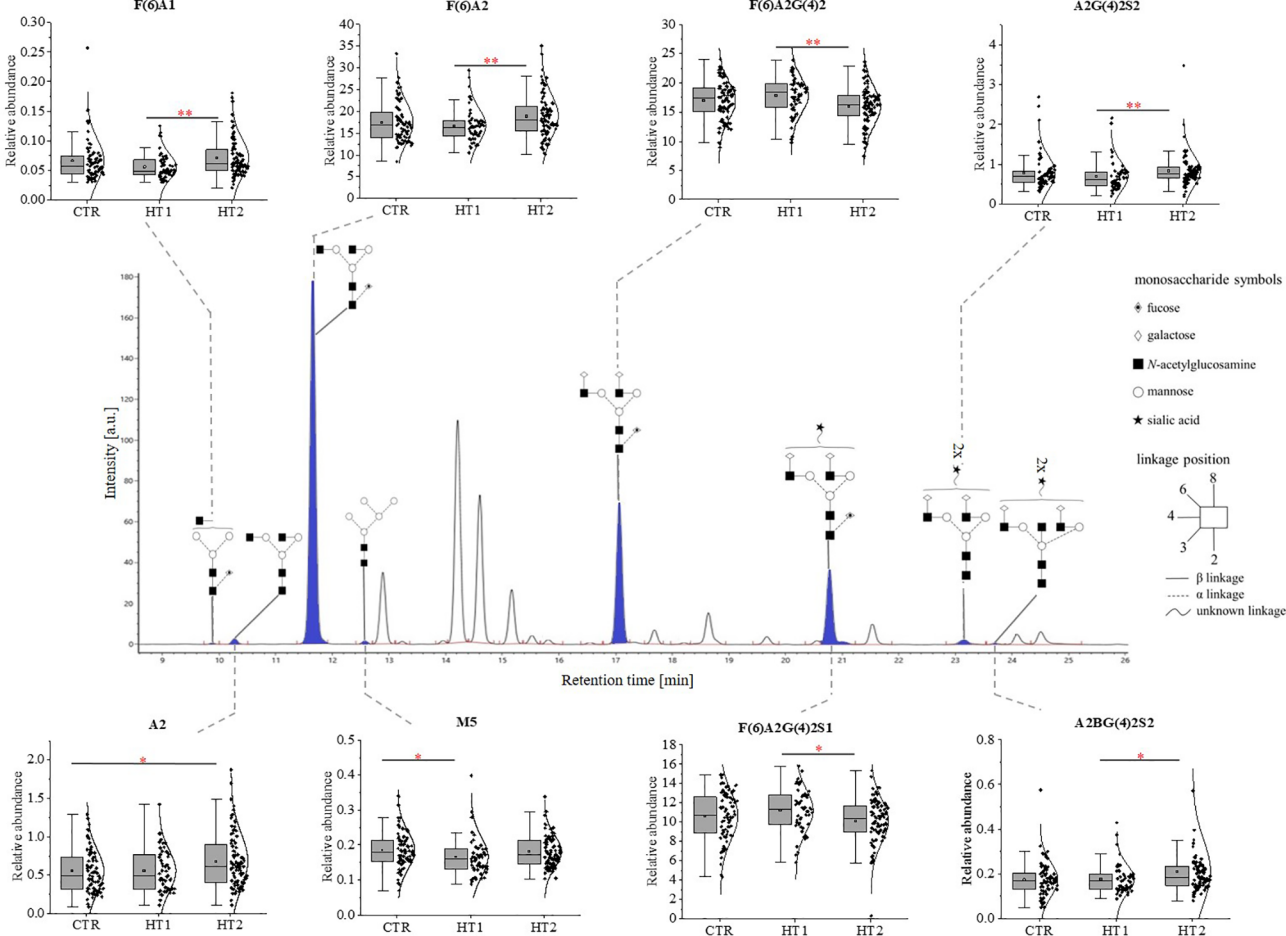
The changes in the content of IgG *N*-glycans from patients with Hashimoto’s thyroiditis patients (HT2, n = 113) and the donors with the elevated IgG anti-thyroglobulin (TgAb) and/or anti-thyroperoxidase (anti-TPO) levels without symptoms of hypothyroidism (HT1, n = 68), and the control group (CTR, n = 90). The chromatograms obtained by UPLC-MS from the separation of IgG *N*-glycans were manually integrated into 25 glycan peaks (GPs) and *N*-oligosaccharide structures were assigned based on GU values and m/z ratios using Waters UNIFI scientific information system software. *N*-glycan structures in Oxford notation were drawn in Sugar Bind software (https://sugarbind.expasy.org/). GPs with statistically significant differences in the content of sugar structures between the study groups (HT1, HT2) and the control group (CTR) are indicated in blue. Statistical analysis was performed using the Kruskal-Wallis non-parametric test, p < 0.05 (*) and p < 0.01 (**). A1-2, number of antennas; B, bisecting *N*-acetylglucosamine; F, fucose; G, galactose, M, mannose; S, sialic acid.

The structure and the amount of *N*-glycans released enzymatically from IgG molecules were analysed by the UPLC-MS method. PNGase F digested *N*-glycans were fluorescently labeled with 2-AB. The obtained resulting chromatograms were integrated into 25 glycan peaks. *N*-oligosaccharide structures were assigned based on the GU values and m/z ratios using Waters UNIFI scientific information system software (**Figures 2**, **3** and **Supplementary Table 1**). The chromatographic pattern of resolved glycans with dominant GP3 was similar for all analysed groups. The identified *N*-glycans were predominantly partially core-fucosylated diantennary complex-type species, and sialylated structures, some of them with bisecting GlcNAc residue. Based on the relative peak intensity, expressed as a percentage of the area of a given GP, statistical analysis was performed using the Kruskal-Wallis nonparametric test with a significance level of p<0.05.

### 3.1 Immunosuppressive Treatment Affects IgG *N*-Glycosylation in Graves’ Disease

The statistically significant differences in IgG *N*-glycan profiles between patients with Graves’ disease before and during immunosuppressive therapy and healthy donors were demonstrated for the twelve out of 25 GP-matched *N*-glycan structures (**Figure 2**). A content of agalactosylated structures with proximal fucose (F(6)A1, F(6)A2, F(6)A2B) and oligomannose (M5) increases in GD (all structures) and GD/T (F(6)A2B and M5) compared with CTR. Statistically significant quantitative differences were also found in galactosylated (A2G(4)2) and fucosylated (F(6)A2[6]BG(4)1) *N*-oligosaccharides with their up-regulation in GD/T patients compared to GD. In contrast to the above-mentioned changes in the case of *N*-glycans with a bisecting GlcNAc (A2BG(4)2), α1,6-fucosylated (F(6)A2BG(4)2), monosialylated (A2G(4)2S1 and F(6)A2G(4)2S1), and disialylated (A2G(4)2S2) structures we observed their statistically significant down-regulation in GD (all structures) and GD/T (A2G(4)2S2) relatively to CTR.

**Figure 4:**
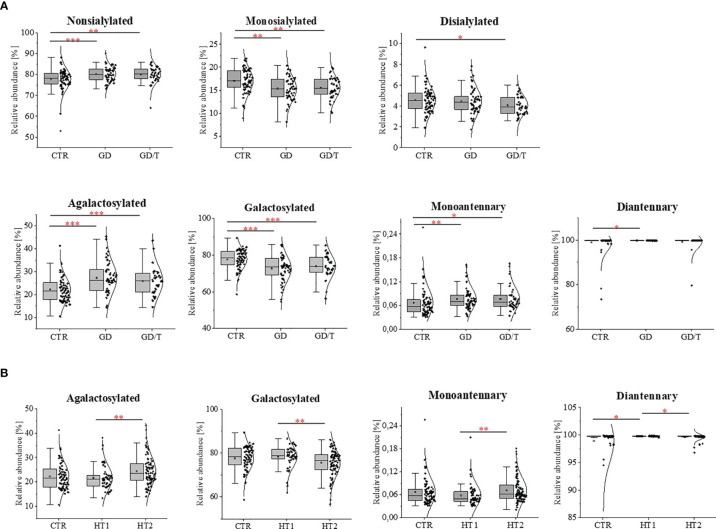
IgG *N*-glycosylation changes in sera of **(A)** the patients with Graves’ disease before (GD, n = 62) and after (GD/T, n = 47) stabilization of TSH level, **(B)** the subjects with the elevated IgG anti-thyroglobulin (TgAb) and/or anti-thyroperoxidase (anti-TPO) levels (HT1, n = 68), Hashimoto’s disease patients (HT2, n = 113) (study groups) relative to the healthy donors (CTR, control group, n = 90). Quantitative comparison of derived glycan traits (nonsialylated, monosialylated, disialylated, galactosylated, agalactosylated, monoantennary, diantennary) between control and study groups was performed by Kruskal-Wallis test assuming a significance level for p < 0. 05 (*), p < 0.01 (**), and p < 0.001 (***).

The effect of *N*-glycosylation on the development of AITD compared with CTR after UPLC-MS analysis was further verified by performing statistical analysis with the Kruskal-Wallis test for glycan groups (non-sialylated, monosialylated, disialylated, non-fucosylated, fucosylated, nongalactosylated, galactosylated, monoantennary, and diantennary). The results showing statistically significant differences are presented in **Figure 4**. Non-sialylated and monoantennary glycans showed an increase in both GD groups compared with healthy volunteers, whereas diantennary structures showed an increasing trend in GD compared with CTR. Statistically significant decreases were shown for monosialylated (in GD and GD/T), and disialylated (in GD) glycans compared with healthy subjects. The relative content of galactosylated structures was lower in GD while in GT/T this group of glycans normalized partly which was related to a decline of agalactosylated structures. The reduced IgG galactosylation was accompanied by an increase of agalactosylated structures in GD (**Figure 4A**).

### 3.2 The Severity of Inflammation in Hashimoto’s Thyroiditis Is Accompanied by Changes of IgG *N*-Glycosylation

Statistically significant quantitative differences were also found for 8 *N*-oligosaccharide structures between patients without hypothyroidism, patients with HT after L-thyroxine treatment, and healthy volunteers (**Figure 4**). For agalactosylated (F(6)A1, A2, F(6)A2), mono- (A2G(4)2S1) and disialylated (A2BG(4)2S2) structures, an increase was shown in HT2 compared to CTR (A2, A2BG(4)2S2) and HT1 (other structures). In contrast, some fucosylated (F(6)A2G(4)2) and monosialylated (F(6)A2G(4)2S1) structures were increased in HT1 relative to HT2. HT1 also indicated a statistically significant decrease in oligomannose-type *N*-glycan (M5) compared to CTR.

A statistically quantitative increase in the content of monoantennary oligosaccharides was observed in HT2 compared to HT1. The amount of diantennary sugar structures was significantly higher in HT1 compared to HT2 and CTR. We identified also a relationship between the alterations of galactosylated and agalactosylated *N*-glycans in HT patients, parallel with the lowering of galactosylation, agalactosylated structures increased in HT2 *vs*. HT1 donors (**Figure 4B**).

### 3.3 ST6Gal1 Is Active in AITD Patients’ Sera and Can Modify IgG Sialylation

**Figure 5:**
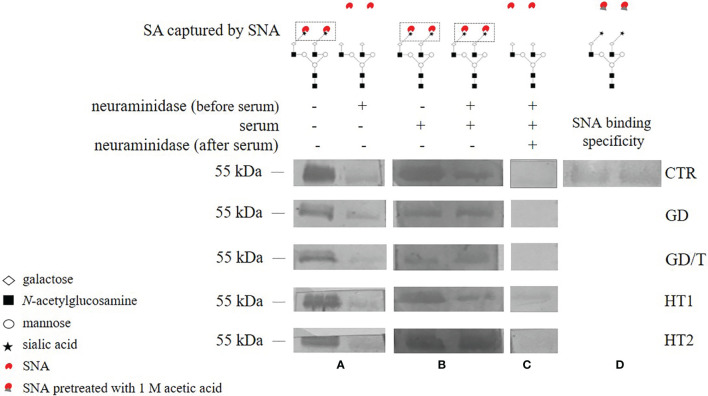
The activity of serum ST6Gal1 assessed by SNA lectin blotting on IgG heavy chain *N*-glycans. IgGs isolated from the sera of the patients with Graves’ disease before (GD) and after (GD/T) TSH normalization, the subjects with the elevated anti-thyroglobulin (TgAb) and/or anti-thyroperoxidase (TPO) antibodies (HT1), Hashimoto’s thyroiditis patients (HT2), and healthy volunteers (CTR, control group) were used. **(A)** IgG heavy chain sialylated (neuraminidase -) and desialylated by neuraminidase from *Arthrobacter ureafaciens* (neuraminidase +), both untreated with serum, **(B)** IgG heavy chain sialylated by serum ST6Gal1 from control and study groups after desialylation by neuraminidase, **(C)** IgG heavy chain redesialylated after serum ST6Gal1 sialylation performed to verified the reversibility of ST6Gal1 sialylation, **(D)** control of SNA binding specificity verified by lectin blotting with SNA preincubated with 1 M acetic acid. More details are described in the section Materials and Methods.

Due to the changes of mono- and disialylated IgG *N*-glycans in both HT and GD sera detected by UPLC-MS analysis (**Figure 4**), we evaluated an activity of ST6Gal1 in sera of AITD patients and healthy individuals using SNA lectin blotting method described by Jones et al. (47) with some modifications. We observed a positive reaction of α2,6-sialylated *N*-glycans on the heavy chain with SNA for IgG isolated from control and AITD sera. Desialylation of IgG using the neuraminidase with a wide specificity resulted in an almost complete attenuation of the reaction with SNA (**Figure 5A**). A weak signal observed for neuraminidase + samples (**Figure 5A**) was a result of non-specific SNA binding to IgG polypeptides as determined by the reaction with SNA preincubated with acetic acid which abolishes SNA binding to SA (**Figure 5D**). Incubation of desialylated IgG heavy chain with human sera restored SNA-positive reaction which resulted from re-sialylation of IgG by ST6Gal1 present in donor sera (**Figure 5B**). To verify reversibility of this sialylation on PVDF membrane-bound IgG heavy chain we used again neuraminidase to remove SA attached by serum ST6Gal1 which led to an almost complete loss of the reaction with SNA (**Figure 5C**).

The detailed characteristic of IgG *N*-glycome obtained by UPLC-MS showed the statistically significant quantitative differences in sugar structures in the course of immunosuppressive treatment of GD patients, and during the development of thyroiditis. The obtained results allow us to support the previous observation that IgG *N*-glycosylation is a particularly dynamic process, reflecting the course of the diseases, and conclude that IgG *N*-glycan alterations accompany the inflammatory processes in AITD and the applied therapy. The changes occur early in the development of the disease when the synthesis of autoantibodies is triggered, and the thyroid structure has not yet undergone the destruction that accompanies chronic inflammation at later stages of the disease. Methimazole therapy significantly changes the structure of the IgG glycome in patients with GD. We have also shown that ST6Gal1, responsible for the attachment of α2,6-SA, is active in the sera of AITD patients and healthy donors and has the potential to modify IgG sialylation independently from the classical pathway of cellular glycosylation.

## 4 Discussion

The research area that has been little addressed so far is the glycoimmunobiology of AITDs. Changes in *N*-glycosylation of immune system molecules in AITDs have only been analyzed concerning serum proteins present in the context of differences between healthy controls and patients (40, 55, 56), while the impact of treatment and the severity of thyroiditis on protein glycosylation has never been focused on. Previous studies have shown that the sialylation and core fucosylation of TgAb *N*-glycans in Hashimoto’s thyroiditis patients were lowered compared to GD donors (56), but elevated in HT compared to healthy individuals (57). Also, oligomannose type structures in TgAb antibodies were increased in HT versus the control group (57). In contrast, our recent study has demonstrated the reduced core fucosylation of IgG in AITD patients compared to healthy donors which correlated of the reduced Fuc content with serum anti-TPO titers (40). On the other hand, the study of Ząbczyńska et al. revealed the up-regulation of disialylated, diantennary complex *N*-glycans and monosialylated, triantennary structures in IgG-depleted sera from patients with HT compared to healthy donors (55).

To explore the glycoimmunology of AITD more deeply, in the present study, we focused on changes in IgG *N*-glycan structures in donors with Graves’ disease before (GD) and after (GD/T) TSH normalization as the result of thyrostatic and immunosuppressive therapy, patients with the elevated titer of antithyroid autoantibodies (HT1), and subjects with Hashimoto’s thyroiditis treated with L-thyroxine (HT2) in comparison to healthy volunteers (CTR).

Our present results are partially in accordance with the previous one by Martin et al. (40). Obtaining full compliance of the results of glycosylation analysis seems to be impossible, because of diverse monosaccharide composition, asymmetric glycosylation in both Fc glycosylation sites, *N*-glycosylation of Fab variable regions, and variant glycosylation of IgG1-4 subclasses, which result in a huge heterogeneity of IgG glycome in a given person (4). It means that drawing reliable conclusions requires verification of the results for various populations and analysis for large study groups.

### 4.1 Methimazole Therapy Impact on IgG *N*-Glycosylation in Graves’ Diseases

The effect of immunosuppressive drugs used in the treatment of autoimmune diseases on *N*-glycosylation of IgG and other serum proteins has been demonstrated in previous studies. This issue has been the most explored in RA, the autoimmune disorders with the best characterized IgG *N*-glycan alterations. It is well documented that one of the effects of immunosuppressive agents administrated in RA is the altered IgG glycosylation reversing the antibody activity towards the anti-inflammatory response (48–51, 58).

GD patients recruited to our study were treated with methimazole (1-methyl-2-mercaptoimidazole; Polish brand names: Thyrozol or Metizol), which affects thyroid hormone synthesis as thyrostatics. Methimazole was shown also to have an immunosuppressive effect resulting from a decrease of TRAb serum level, triggering of intrathyroidal T cell apoptosis, reduction of HLA class II expression as well as up-regulation of circulating Tregs and reduction of the number of Th cells, NK, and activated intrathyroidal T cells (59–61). TRAb concentration in the sera of methimazole-treated GD patients was partly reduced (**Table 1** and **Figure 1A**), which confirms the immunosuppressive activity of this antithyroid agent. Taking into account that the blood was taken in the relatively short term after stabilization of TSH levels, it can be assumed that in the longer term TRAb titer was further stabilized.

The normalisation of TRAb level in GD/T (**Table 1** and **Figure 1A**) was accompanied by the altered intensity of the individual UPLC-MS peaks to which agalactosylated (F(6)A2), monogalactosylated (F(6)A2(5)BG(4)1), and digalactosylated (A2G(4)2, F(6)A2G(4)2 and F(6)A2BG(4)2) glycans were assigned. The amount of both digalactosylated *N*-glycans was up-regulated while the content of agalactosylated structure was reduced as the result of methimazole therapy in GD patients (**Figures 2**, **4A**). This part of our observations is following the previous experiments on IgG *N*-oligosaccharides from RA patients undergoing treatment with methotrexate and infliximab. In addition to suppressing inflammatory processes confirmed by a reduced level of C-reactive protein (CRP) (58), both antirheumatic drugs were shown to enhance IgG galactosylation (48–51, 58). In turn, in immune thrombocytopenia galactosylation of IgG1 and IgG4 subclasses in the blood of anti-CD20 monoclonal antibody (rituximab)-treated patients was slightly reduced, while IgG1, IgG2/3, and IgG4 showed the higher content of *N*-glycans with bisected GlcNAc (62). Therefore we can conclude that the effect of immunosuppressive therapy on *N*-glycosylation depends on the applied therapeutic agent and autoimmune disorder.

Apart from the significantly increased galactosylation of *N*-glycans in inflammatory arthritis patients treated with anti-TNF IgG, Collins et al. observed an up-regulation of *N*-glycan core-fucosylation (58), which is well known to contribute to IgG anti-inflammatory activity (6). Our study demonstrated that the amount of IgGs with core-fucosylated structures F(6)A2(6)BG(4)1, F(6)A2G(4)2, and F(6)A2BG(4)2 is higher in GD patients after TSH normalization as the result of methimazole therapy (GD/T) in relation to the untreated donors (**Figure 2**) which can also contribute to attenuating of the immune response.

According to the literature data, reduced sialylation favors the pro-inflammatory properties of IgG (63, 64). Our UPLC-MS analysis showed that the sialylation status of IgG was not affected by methimazole treatment. The intensity of the sialylated *N*-glycans was not altered in GD patients during immunosuppressive therapy in relation to the state before this drug implementation (GD/T *vs*. GD) (**Figure 2**). However, the entire pool of IgG monosialylated and disialylated *N*-oligosaccharides was significantly reduced in both GD groups in comparison to healthy donors (**Figure 4A**). Sialic acid is terminally linked to Gal residues in IgG *N*-glycans, and its attachment requires the presence of galactosylated structures. We presently show that galactosylation of IgG is normalized during methimazole administration (**Figure 4A**) while *N*-glycans are still undersialylated in relation to healthy donors (**Figure 2**), which can be an effect of too short drug administration. To resolve this, studies on a long-term treatment cohort would be necessary.

Methimazole therapy partially reversed the changes of IgG *N*-glycosylation by the up-regulation of galactosylation and reduction of agalactosylation (**Figure 4A**). Down-regulated galactosylation is well known to promote the proinflammatory potential of IgG (34). Without detailed functional studies, we can only speculate that this effect of anti-inflammatory treatment may reduce clinical symptoms of the disease also by affecting galactosylation of antibodies in GD.

Due to the documented activity of serum ST6Gal1 from AITD patients on desialylated IgG *N*-glycans (**Figure 5**), we suppose that this serum sialyltransferase may contribute to IgG sialylation in the bloodstream in thyroid autoimmunity. SNA lectin blotting used in our study to assess ST6Gal1 activity provided the qualitative data but did not allow for quantitative analysis and determination of possible differences between groups. A quantitative assessment of the effects of ST6Gal1 activity would be interesting to interpret the UPLC-MS results, and is worth pursuing in future studies. Anti-inflammatory activity of IgG sialylation is also considered as a therapeutic strategy in terms of the usage of intravenous immunoglobulins (IVIGs) for the treatment of autoimmune diseases (65, 66). Pagan et al. used the mechanism of extracellular sialylation to target IgG *N*-glycan remodeling. They confirmed the attaching of SA by the extracellular enzyme to Fc of IgGs deposited at sites of inflammation. The activity of recombinant human galactosyltransferase B4GALT1 and ST6Gal1 on a single Fc fragment of IgG1 attenuated inflammation in a K/BxN arthritis model. The increased IgG sialylation resulted from *in vivo* administration of a soluble ST6GAL1 converted IgG activity into anti-inflammatory in autoimmune disease (67).

In the previous study, we have also demonstrated the altered glycosylation on human leukocytes activated *in vitro* in a two-way mixed leukocyte reaction (MLR) in the presence of two immunosuppressive agents commonly used to induce immune tolerance after organ transplantation: cyclosporin A (CsA), an inhibitor of calcineurin, and rapamycin (Rapa), which blocks mammalian target of rapamycin mTOR. Oligomannose/hybrid-type *N*-glycans on human leukocytes in MLR model were significantly down-regulated by CsA, while the synergistic action of both immunosuppressive drugs enhanced the amount of these structures on leukocyte proteins (68).

Based on the previous literature data and the presently obtained results, it seems that immunosuppressive agents-triggered changes of glycosylation are a wider phenomenon, which can concern drugs with various mechanisms of action, and different targeted proteins. Functional consequences of the observed changes in IgG *N*-glycosylation isolated from methimazole-treated patients for Graves’ disease course need further study.

### 4.2 Altered IgG *N*-Glycosylation in Autoimmune Thyroiditis May Contribute to Thyroid Destruction

The crucial role of Asn297-linked *N*-glycans attached to IgG Fc fragment in ADCC and CDC has been demonstrated on various research models (69), and was shown to be important in autoimmune disease development (70, 71), including thyrocyte destruction in Hashimoto’s thyroiditis (72). Forming of immune complexes of IgG autoantibodies with self-antigens gathers innate immune effector cells, like NK and myeloid cells, which express Fcγ receptors (FcγR) and/or recruits complement proteins. Activation of effector cells and complement cascade results in target cell apoptosis and finally tissue damage (34, 73).

Martin et al. detected the reduced level of core Fuc in IgG *N*-glycans from AITD subjects and depletion of antenna fucosylation in peripheral blood mononuclear cells (PBMCs) isolated from the whole blood of HT patients (40). The current analysis showed that the content of two core-fucosylated IgG structures (F(6)A2G(4)2 and F(6)A2G(4)2S) was decreased in Hashimoto’s thyroiditis patients, while the level of F(6)A2 *N*-glycan assigned the most abundant UPLC peak was up-regulated in these patients (HT2) compared to the donors with the higher level of anti-Tg/anti-TPO (HT1) (**Figure 3**). In effect, the group analysis did not show statistically significant changes in the whole pool of core-fucosylated *N*-glycans between the patients with thyroiditis at different stages of the disease severity and healthy subject (**Figure 4**). Taking into account the significantly different number of the recruited participants in both studies, not complete reproducibility of the obtained results seems to be understandable.

Core-fucosylation of immune system proteins, including IgG, plays an important role in their activity. Alterations in IgG core-fucosylation are, right after galactosylation, one of the most common modifications detected in inflammatory diseases, including autoimmune diseases (37). Based on the previous results which demonstrated that core-fucosylation impeded FcγRIIIA binding and inhibited ADCC (47), we can speculate that the reduced core-fucosylation of IgG *N*-glycans in AITD described by Martin et al. (40), and the currently observed decrease of the two core-fucosylated structures in HT2 (**Figure 3**) may contribute to inflammation and thyroid tissue damage in the course of Hashimoto’s thyroiditis.

Sialic acid is a component of glycans crucially important in the regulation of immune glycoprotein function, due to its negative charge, and terminal localization in oligosaccharide structures. Autoimmunity is usually accompanied by down-regulation of IgG sialylation (74, 75). The reduced sialylation of IgG1 and IgG2 occurs in granulomatosis with vasculitis (GPA) (64). Kemna et al. showed that the level of total IgG sialylation has a prognostic value in GPA relapse, as the SA content of IgG1 in patients with granulomatosis decreased during relapse and remains unchanged in remission (63). Collins et al. demonstrated a decrease in sialylated triantennary *N*-glycans in inflammatory arthritis patients, strongly correlated with reduced CRP level in the silenced inflammatory process (73).

Our recent study has demonstrated more intensive thyrocyte lysis in the presence of IgG isolated from HT patients than from healthy donors, which resulted from the higher anti‐TPO content in the whole IgG pool of HT donors and the altered IgG *N*-glycosylation in HT autoimmunity (72). The present analysis demonstrated that the content of F(6)A2G(4)2S1 structure, the most intensely sialylated *N*-glycan in IgG glycoprofile, was lowered in Hashimoto’s thyroiditis in comparison to HT1 group (**Figure 3**), which may explain the results obtained in the previous functional analysis performed with IgG from HT patients in the *in vitro* model (72). The study by Ząbczyńska et al. indicated also that IgGs with desialylated *N*-glycans were more potent to induce ADCC in human thyrocytes (72).

In summary, *N*-glycans are involved in fundamental cellular and molecular processes that stimulate and inhibit immune system pathways. Detailed characterization of the IgG *N*-glycans obtained by UPLC-MS revealed statistically significant quantitative differences in sugar structure during immunosuppressive treatment of GD patients and during the development of Hashimoto’s thyroiditis. The results show that changes in IgG *N*-oligosaccharides contribute to the development of inflammation in autoimmune thyroid diseases. These changes begin in the early stages of the disease, where autoantibodies are overproduced, but hypothyroidism and thyroid gland destruction, which are associated with later stages of pathology, are not observed. The use of immunosuppressive therapy significantly alters the process of *N*-glycosylation in patients with Graves’ disease. Further studies are needed to evaluate the changes in IgG *N*-glycosylation in AITD to see how relevant it is to determine the contribution of altered oligosaccharide content to antibody-mediated autoimmunity in these autoimmune diseases. It is also worth noting, that similarly as in the case of vast majority of glycomic studies of this kind, we compare only relative quantities of glycan structures in tested samples. By its nature, the relative content of a given sugar structure in a sample depends not only on the increase or decrease in its expression, but also on the simultaneous quantitative changes of other glycans. Being aware of these limitations, we used this approach in the described studies and we believe that it is, however, a specific description of the phenotype of the patients and enables comparative analysis. On the one hand, it gives less room for interpretation of the biological contexts and mechanisms causing the observed changes, but on the other hand, when applied according to the same criteria for all analysed samples, it can be a useful parameter for patient stratification.

## 5 Concluding Remarks

*N*-glycosylation of immune proteins fundamentally affects their structure, half-life, activity and interaction with protein partners on other cells or soluble ones present in body fluids (76). For a long time the sugar part of glycoproteins has been considered only as an insignificant decoration, until the results of glycoanalysis obtained for IgG showed, how essential sugar structures are for the proper biochemical properties, and biological activity of this molecule. Thanks to the development of new research technologies, we are increasingly aware of the great role played by glycans under physiological conditions. In turn, determining how structure of oligosaccharides is remodeled in human pathologies, and what the consequences of these changes are for the course of disease has not been well studied (8, 9, 12). *N*-glycosylation of IgG in AITDs was relatively poorly studied, although HT and GD are among the most common autoimmune diseases. Our present study provides new data on IgG remodeling in the course of AITD. The questions are how the observed changes of IgG *N*-glycosylation affect its activity, and what consequences these oligosaccharide modifications have for the development of the disease or, in the case of treatment, for the recovery or the reduction of symptoms, remain to be answered in further studies.

## Data Availability Statement

The original contributions presented in the study are included in the article/**Supplementary Material**. Further inquiries can be directed to the corresponding author.

## Ethics Statement

The studies involving human participants were reviewed and approved by Bioethics Committee of the Jagiellonian University in Kraków, Poland. The patients/participants provided their written informed consent to participate in this study.

## Author Contributions

EP designed the study and secured grant funding. GS recruited blood donors. ST and PL-L performed experiments, and analyzed data. ST drafted the manuscript. EP and PL-L revised the manuscript. All authors contributed to the article and approved the submitted version.

## Funding

The study was supported by a grant from the Polish National Science Centre (grant no. 2015/18/E/NZ6/00602).

## Conflict of Interest

The authors declare that the research was conducted in the absence of any commercial or financial relationships that could be construed as a potential conflict of interest.

## Publisher’s Note

All claims expressed in this article are solely those of the authors and do not necessarily represent those of their affiliated organizations, or those of the publisher, the editors and the reviewers. Any product that may be evaluated in this article, or claim that may be made by its manufacturer, is not guaranteed or endorsed by the publisher.

## Supplementary Material

Supplementary Figure 1 Protein profiles of IgG separated by SDS-PAGE under reducing conditions after Coomassie Brilliant Blue (CBB) staining. IgG heavy and light chains are indicated by red boxes. MW, molecular weight marker (Page Ruler Prestained Protein Ladder, Thermo Scientific, 26616).

Supplementary Table 1 UPLC-MS *N*-glycan peaks annotation. Glycan structures were identified in Waters UNIFI Scientific Information System based on glucose unit values (GU) and the exact mass. The Oxford notation was used for glycan names and symbolic representations. Observed GU and observed masses were calculated as averages of all samples analysed. The structures of *N*-glycans in Oxford notation (UOXF) were prepared in Sugar Bind software (https://sugarbind.expasy.org/).
