# Supplementary figures and images for: Changes of IgG N-Glycosylation in Thyroid Autoimmunity: The Modulatory Effect of Methimazole in Graves’ Disease and the Association With the Severity of Inflammation in Hashimoto’s Thyroiditis
Source: Front Immunol. 2022 Mar 15;13:841710. doi: 10.3389/fimmu.2022.841710 (PMC8965101; doi:10.3389/fimmu.2022.841710)

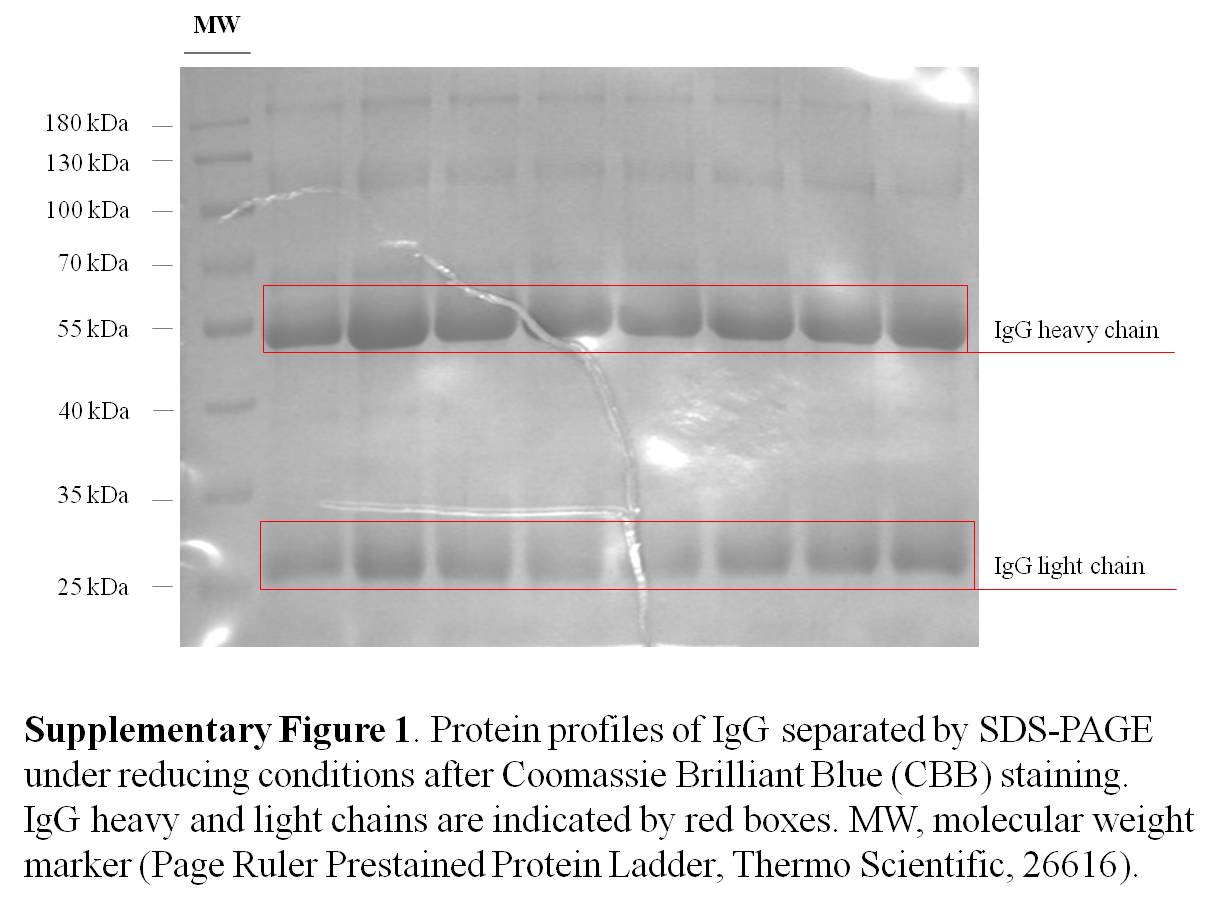

Supplement: Supplementary Figure 1 — Protein profiles of IgG separated by SDS-PAGE under reducing conditions after Coomassie Brilliant Blue (CBB) staining. IgG heavy and light chains are indicated by red boxes. MW, molecular weight marker (Page Ruler Prestained Protein Ladder, Thermo Scientific, 26616). [file Image_1.jpg]
